# Process parameter sensitivity investigation on the reaction crystallization production of K_2_SO_4_ with salt lake leonite ore

**DOI:** 10.1039/c9ra06662d

**Published:** 2019-09-30

**Authors:** Hang Chen, Min Zhu, Xingfu Song, Jianguo Yu

**Affiliations:** Engineering Research Center of Resource (Salt Lake) Process Engineering, Ministry of Education Shanghai 200237 China xfsong@ecust.edu.cn jgyu@ecust.edu.cn +86-21-64252346 +86-21-64252346; National Engineering Research Center for Integrated Utilization of Salt Lake Resource, East China University of Science and Technology Shanghai 200237 China; Shanghai Institute of Pollution Control and Ecological Security Shanghai 200092 China

## Abstract

This paper mainly focused on the reaction crystallization production of K_2_SO_4_ to support the brine resource development in Western China. The process parameters of material ratio, water addition, agitation rate, and operating temperature were investigated to clarify their sensitivity effects on the objectives of product purity, recovery and crystal size. The results show that the mass ratio of leonite ore to KCl should be close to the operating point of equivalent reaction so that the conversion is complete. Meanwhile, the factors of water addition and temperature have the same influence mechanism on the K_2_SO_4_ production by changing the solubility equilibrium. Small water addition and low temperature are suggested for a high potassium recovery. However, they have critical values to ensure the complete dissolution of the raw materials. The intensified agitation will reduce the crystal size significantly, whereas it has no effect on the purity and recovery provided the operating time is enough. Hence, the agitation rate should be as small as possible for a large particle product on the preconditions of acceptable operating time to reach the system equilibrium. Based on the optimized operation, the product has first grade quality in bench-scale experiments. Related results provide important references for the design and optimization of industrial K_2_SO_4_ production.

## Abstract 2

Reaction crystallization process optimization for the production of K_2_SO_4_ with low-grade salt lake leonite ore in Qinghai, China.

## Graphical abstract

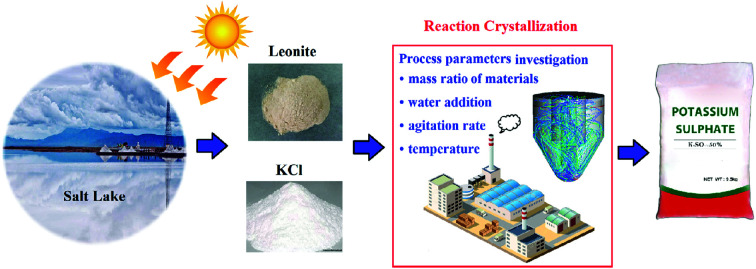

## Introduction

As one of the three main fertilizer elements (N, P, K), potassium is of great significance to agricultural production and food security.^1^ Nowadays, China has been the fourth largest potash fertilizer manufacturing country in the world. Its annual output of potash fertilizer reaches around 10 million tons.^2^ However, as the world potash fertilizer industry, it faces the problem of simple product structure.^3^ Due to the resource constraints, nearly 70% of the potash fertilizer in China is KCl. The production of three main chloride-free potash fertilizers,^4^ namely, K_2_SO_4_, KH_2_PO_4_ and KNO_3_, is still far away from the market requirement.

It is well known that K_2_SO_4_ is a high-efficiency sulfur–potassium compound fertilizer, which is essential for some special cash crops such as fruit trees and tobacco.^5^ It is also an important substrate material for preparing phosphors.^6,7^ In the past, K_2_SO_4_ known as a processing-type product was mainly manufactured by converting KCl with reaction conversion methods such as the Mannheim process, Glaserite process and Phosphogypsum process.^8–10^ Among them, the Mannheim process is the most widely used one. The raw materials of KCl and H_2_SO_4_ are used to prepare K_2_SO_4_ based on the principle of metathesis reaction. Although new technologies were proposed to intensify the reaction conversion production, it is easy to cause the problems of environmental pollution and equipment corrosion due to the generation of by-product HCl.^11–14^ This becomes the main limitation factor for the actual application of the Mannheim method. Recently, thanks to the discovery of magnesium sulfate sub-type salt lake brine resource in Western China,^15,16^ the production of resource-type K_2_SO_4_ production has made a great progress. SDIC Xinjiang Lop Nur Potash Co. Ltd. (SLNP) has been the biggest K_2_SO_4_ production company in the world with the annular output around 1.6 million tons.^17^ The traditional processing-type product is replaced gradually by it in the K_2_SO_4_ market. Based on this fact, the high-efficiency production of resource-type K_2_SO_4_ is becoming the main development direction of the K_2_SO_4_ industry.

At present, the magnesium sulfate sub-type salt lake brine resource for producing resource-type K_2_SO_4_ is mainly distributed in Xinjiang and Qinghai Provinces.^15,16^ The resource grade in Qinghai is relatively low so that current K_2_SO_4_ resource development is mainly concentrated in Xinjiang represented by Lop Nur salt lake. Take SLNP as an example, the K_2_SO_4_ production includes three main procedures. Specifically, potassium mixed salt and carnallite will be harvested firstly from the salt lake brine through the sun-dried process. They are used to produce schoenite and KCl with the main processes of decomposition conversion and flotation separation. Then, the K_2_SO_4_ can be manufactured by the reaction crystallization conversion between schoenite and KCl.^18–21^ The main processes of KCl preparation and K_2_SO_4_ production can be summarized briefly as the following equations.

1 KCl·MgCl_2_·6H_2_O(s) + *n*H_2_O(l) = KCl(s) + MgCl_2_(l) + (*n* + 6)H_2_O(l)

2 K_2_SO_4_·MgSO_4_·6H_2_O(s) + 2KCl(s) + *n*H_2_O(l) = 2K_2_SO_4_(s) + MgCl_2_(l) + (*n* + 6)H_2_O(l)

where s and l in the brackets represent the phase states of solid and liquid, respectively. KCl·MgCl_2_·6H_2_O and K_2_SO_4_·MgSO_4_·6H_2_O are carnallite and schoenite, respectively. There is no doubt that the reaction crystallization conversion between schoenite and KCl is the key step for the K_2_SO_4_ production. For this production process, related parameters optimization is always an important research topic. Liu^22^ once discussed the effects of operating temperatures on the purity, recovery and size of the crystal product. Different temperature control schemes were proposed considering the apparent temperature difference in different seasons. Li *et al.*^23^ investigated the experiment conditions and control parameters such as raw material ratio, water addition, conversion time, operating temperature, agitation intensity for the K_2_SO_4_ production process. The optimized results of 57.54% conversion rate and 43.27% product purity (K_2_O) were acquired. Hu *et al.*^24^ also optimized the reaction conversion process parameters, while their focus was mainly on the index of crystal size. The factors of operating temperature, seed addition, feeding sequence and feeding rate were improved for preparing large K_2_SO_4_ crystal particles. In addition, as the main reference for process intensification, the kinetics of schoenite dissolution and reaction crystallization conversion were investigated by different researchers and corresponding kinetic models were established.^25–30^ Related results support the industrial K_2_SO_4_ production in Xinjiang effectively, however, systematic parameter optimization investigations are still lacking.

Compared with Xinjiang, the K_2_SO_4_ industry in Qinghai is still undeveloped. This is mainly ascribed to the different characteristics of local brine resource. Existing investigations indicated that the composition and impurities in the raw ore may have significant effects on the production process and product quality.^31^ Hence, it is necessary to investigate the process conditions considering the different ore resources. This study mainly concerned on the reaction crystallization production of K_2_SO_4_ with the salt lake resource in Qinghai. The main process parameters of raw material ratio, water addition, agitation rate and operating temperature were investigated for an optimal operation with three main objectives of high purity, high recovery and large crystal size. Related sensitivity results are expected to provide an effective support for the industrial resource development and utilization in Qinghai.

## Experiments

### Experimental materials

**Fig. 1:**
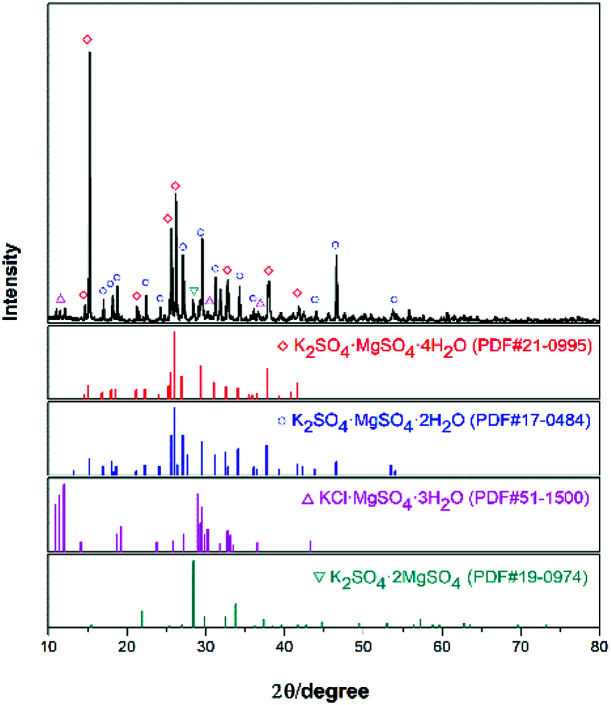
XRD pattern of the leonite ore.

The experimental raw materials mainly include the leonite ore and KCl. The leonite ore was acquired through the sun-dried process of the brine in Qinghai. XRD is a widely used method for resolving the material structure.^32,33^ As shown in Fig. 1, the XRD result of the leonite ore indicates that it includes the main components of K_2_SO_4_·MgSO_4_·4H_2_O and K_2_SO_4_·MgSO_4_·2H_2_O with a small amount of KCl·MgSO_4_·3H_2_O and K_2_SO_4_·2MgSO_4_. Further quantitative analysis was conducted to determine the composition of the raw ore and the results are presented in Table 1. The leonite ore was used directly without any further purification. Meanwhile, analytical pure KCl purchased from Sinopharm Chemical Reagent Co., Ltd was used. The deionized water with the electrical conductivity less than 1 μS cm^−1^ was used in the experiments.

**Table 1:** Composition of the leonite ore

Components	Mg	K	Na	Cl	S	Insoluble
wt%	7.12	19.59	0.82	3.15	16.55	1.02

### Experimental methods

**Fig. 2:**
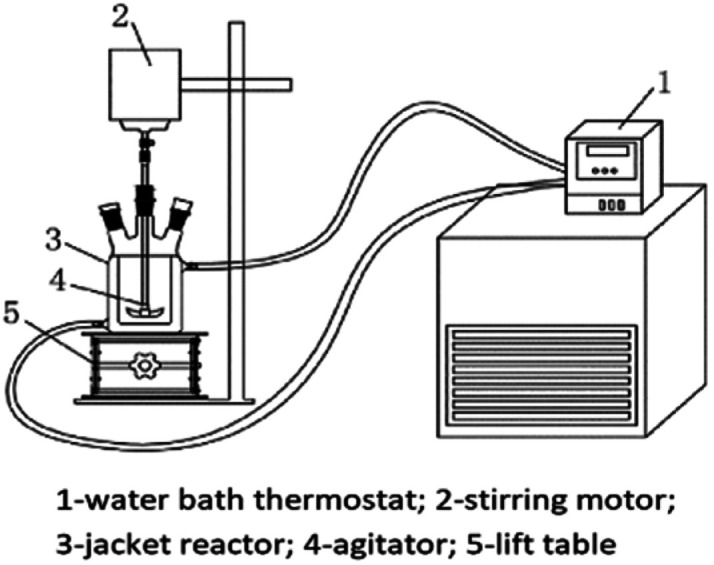
Experimental device for the reaction crystallization production of K_2_SO_4_.

For the reaction crystallization production of K_2_SO_4_, the experimental device is shown in Fig. 2. The reaction conversion and crystallization processes between leonite and KCl were carried out in a preformed jacketed reactor. A mechanical agitator was installed to intensify the material mixing. Besides, the operating temperature was controlled by a water bath thermostat (Shanghai Hengping Scientific Instruments Co. Ltd., DC-2006) with the accuracy of ±0.1 K.

For the specific experiments, the quantities of leonite ore, KCl and deionized water were pre-calculated and weighed by an analytical balance. Considering the slow dissolution kinetic, the leonite was pre-dissolved completely into the deionized water firstly. Subsequently, the weighed KCl was added into the solution, which meant the start of the reaction crystallization conversion. The conversion process was continued for at least 30 min to ensure the complete equilibrium. During this period, the jacketed reactor was maintained at a constant temperature through the water bath thermostat and the agitation rate was fixed. After this, the solid–liquid separation was performed for the mixed materials with the manner of lab-scale vacuum filtration. Then, the liquid phase was sampled for analysis directly, while the solid phase crude product was dried at 105 °C before its quantitative analysis.

### Analytical methods

The two-phase composition analysis mainly includes the contents of K, Mg, Cl and S. Among them, the K content was determined by sodium tetraphenylborate volumetric method. The Mg content was measured by EDTA complexometric titration. The Cl content was titrated with standard silver nitrate solution in the presence of potassium chromate. The element S was existed in the form of SO_4_^2−^ so that its content was measured by gravimetric method with excess barium sulfate solution. In addition, the Malvern laser particle size analyzer (MASTERSIZE 3000, UK) was employed to measure the crystal size distribution of the wet K_2_SO_4_ crude product.

## Results and discussion

### Effects of mass ratio of leonite to KCl on the K_2_SO_4_ production

**Fig. 3:**
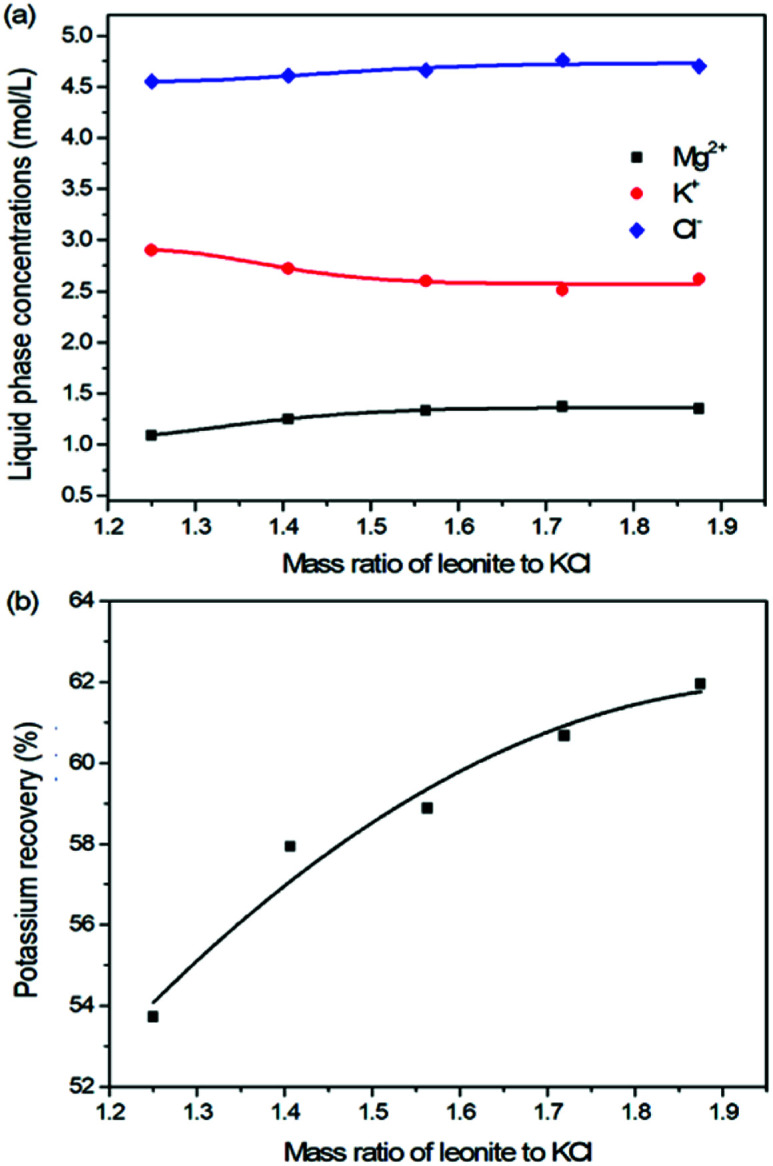
Effects of mass ratio of leonite to KCl on (a) liquid phase ion concentrations; (b) potassium recovery.

**Fig. 4:**
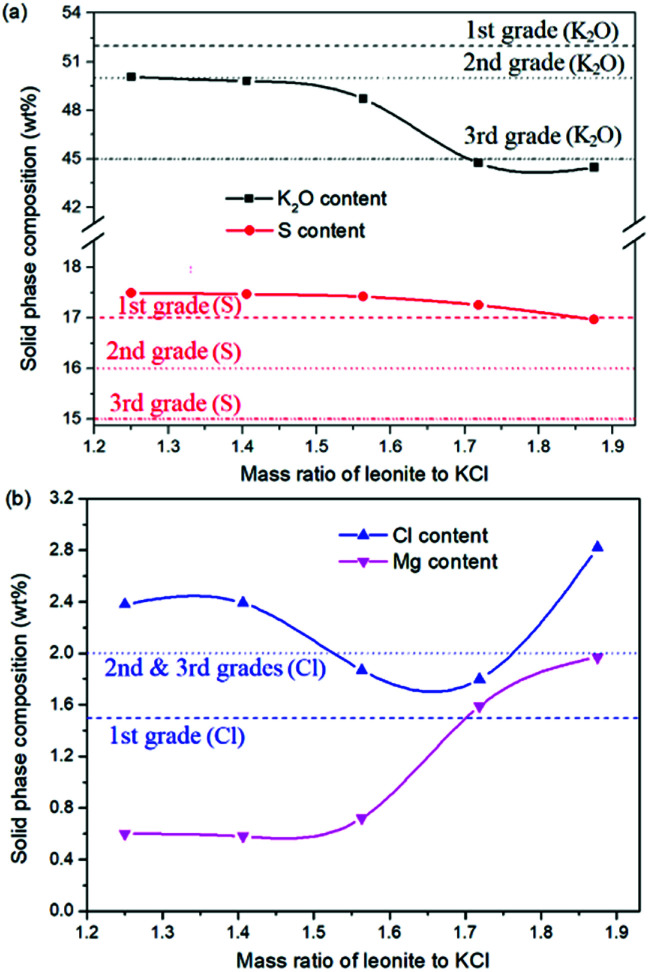
Effects of mass ratio of leonite to KCl on the product composition (a) product purity; (b) impurity content.

**Fig. 5:**
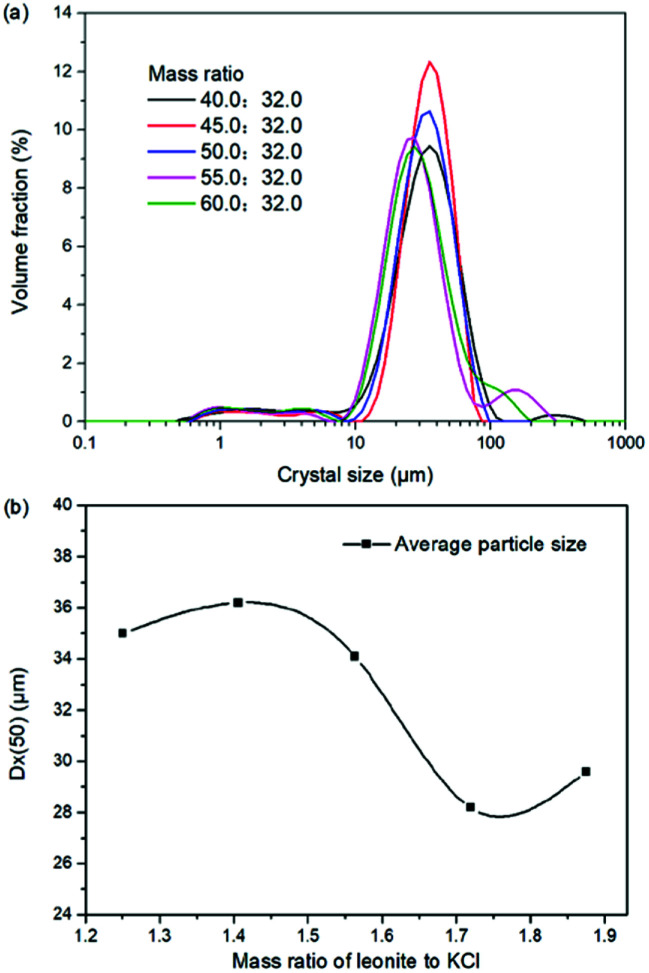
Effects of mass ratio of leonite to KCl on (a) crystal size distribution; (b) crystal averaged size.

The production of K_2_SO_4_ involves the reaction conversion between leonite ore and KCl. Certainly, the mass ratio of the materials is an important parameter for such a process. For this parameter investigation, the quantities of KCl and deionized water were fixed to be 32.0 and 82.5 g, respectively. For an equivalent reaction, the mass of leonite should be 50.0 g according to the raw ore composition. Then, the quantity of leonite ore was changed from 40.0 to 60.0 g to adjust the mass ratio of leonite to KCl. The experiments were conducted at the constant temperature of 298.15 K and the agitation rate was fixed to be 400 rpm. The liquid phase ion concentrations and the dried solid phase composition were determined. Meanwhile, the dried solid phase was weighed further to calculate the potassium recovery. The results are shown in Fig. 3 and 4, while the crystal size analysis results are presented in Fig. 5.

As shown in Fig. 3(a), the liquid phase concentrations of Mg^2+^ and Cl^−^ increase slightly with the increasing mass ratio of leonite ore to KCl, whereas the K^+^ content decreases gradually. This signifies that increasing the proportion of leonite ore is advantageous to improve the reaction conversion rate so that the potassium recovery has the increasing trend as shown in Fig. 3(b). About the solid phase product, the results in Fig. 4(a) indicates that the purity of the K_2_SO_4_ product decreases with the increasing mass ratio of leonite ore to KCl. In others words, the purposes of high product quality and high resource recovery are always conflicting. In general, the decreasing trend of the product purity can be divided into two stages. At the beginning, the contents of K_2_O and S are almost constant with a slight decrease. When the mass ratio of leonite to KCl reaches ∼1.6, the contents have an apparent decline and fall into the second stage. Besides, the variations of impurities in Fig. 4(b) are relatively complicated. The variation trend of the impurity Mg is just the opposite with the one of K_2_O, whereas the content of Cl decreases first and increases later with the increasing mass ratio of leonite ore to KCl. Combing with the results of crystal size as shown in Fig. 5, it can be believed that the variation of impurity Mg is resulted from two potential factors. One is the mother liquor entrainment effect, which depends on the crystal size directly. Specifically, the large crystal size at the low mass ratio of leonite ore to KCl is advantageous to reduce the mother liquor entrainment, while the reduced crystal averaged size at high material ratio is easy to cause the entrainment of Mg. On the other hand, the crystal size distributions at high mass ratio of leonite ore to KCl have the characteristic of dual peaks. This phenomenon indicates that the leonite ore may be undissolved so that the Mg content in the product is increased. By contrast, the variation of impurity Cl has the main difference of its decreasing at the beginning. This is probably attributed to incomplete conversion of KCl.

According to the national standard GB/T 20406-2017, the agricultural K_2_SO_4_ mainly has the purity requirements on the elements of K_2_O, S and Cl. It is classified into three grades based on these purity indexes. As shown in Fig. 4, the critical values of the required indexes were expressed by the dash, dot, dash–dot–dot lines for the first, second and third grades, respectively. It can be find that the product grade is mainly determined by the indexes of K_2_O and Cl, whereas the S content is easy to get the first grade. In the first stage of low mass ratio of leonite ore to KCl, the K_2_O content is closed to the standard of the second grade, however, it is reduced to the third grade in the second stage of high mass ratio of leonite ore to KCl. Combing with the result of Cl content, the material mass ratio of 1.65 (leonite ore 50.0 g, KCl 32.0 g) is considered as the optimal operating point, where the crude K_2_SO_4_ has the product quality closest to the second grade with the acceptable potassium recovery of 58.88%. In other words, the equivalent reaction is the best. It should be noticed that this is a result to balance the indexes of product impurity and potassium recovery. It is independent of the phase equilibrium and crystallization route with their focus on the product yield.

### Effects of water addition on the K_2_SO_4_ production

Expect the mass ratio of leonite to KCl, the water addition is another important raw material factor. As the main solvent for the reaction crystallization conversion, the amount of water addition may have significant effects on the K_2_SO_4_ production through the phase equilibrium regularity. To ensure the complete dissolution and reaction conversion, the minimum addition amount of the deionized water was calculated based on the stable phase equilibrium of the system K^+^, Mg^2+^‖Cl^−^, SO_4_^2−^–H_2_O at 298.15 K. The theoretical minimum water addition was confirmed to be 75.2 g corresponding to the raw materials of 50.0 g leonite and 32.0 g KCl. Then, the investigated water addition was adjusted to be 1.0–1.6 times of the theoretical value. The experiments were conducted at the agitation rate of 400 rpm and constant temperature of 298.15 K.

**Fig. 6:**
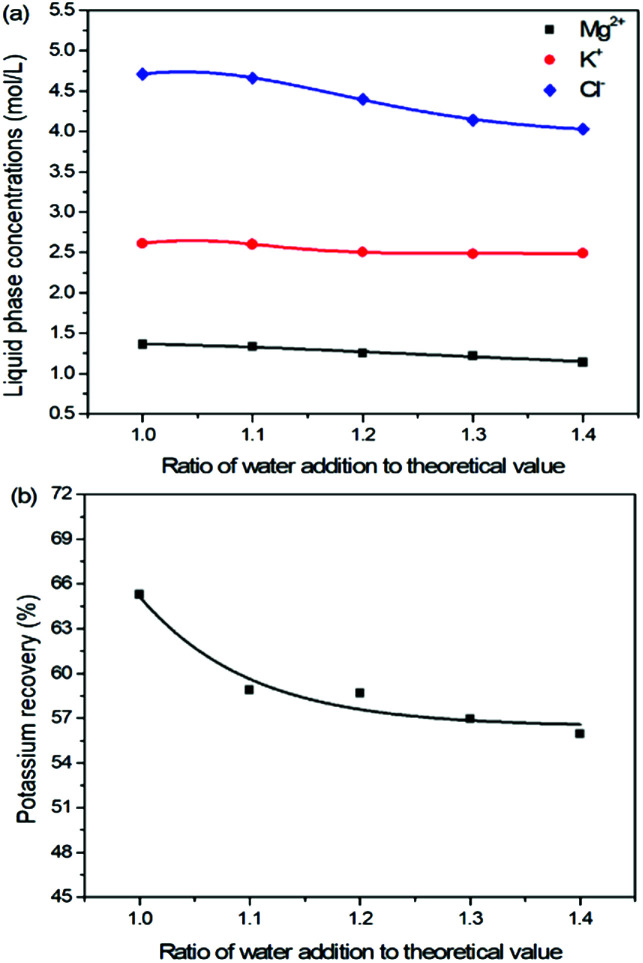
Effects of water addition on (a) liquid phase ion concentrations; (b) potassium recovery.

**Fig. 7:**
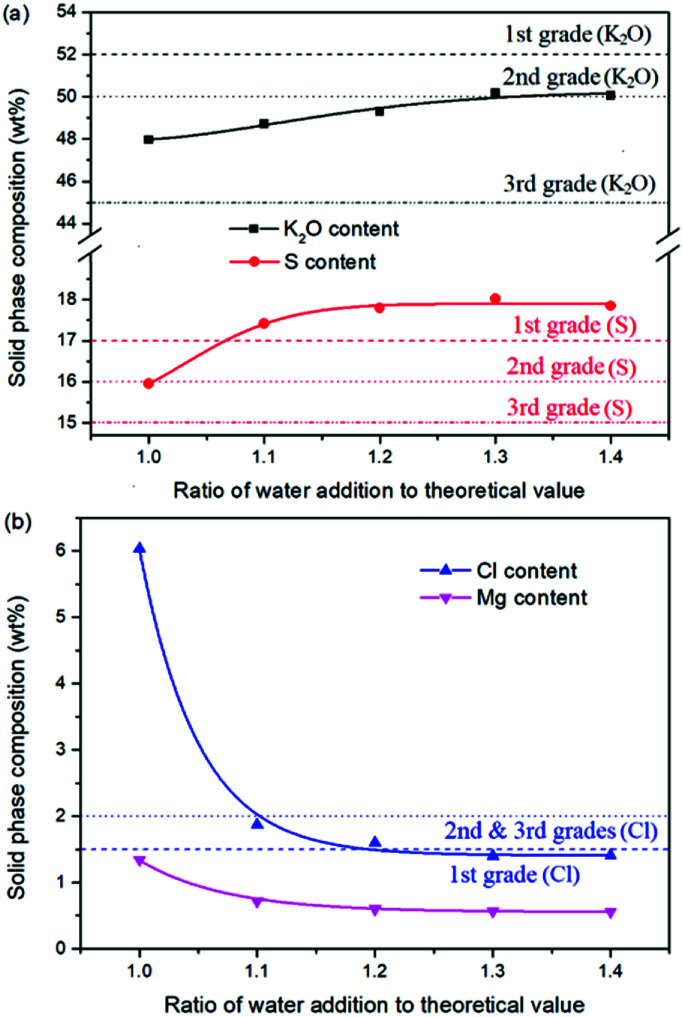
Effects of water addition on the product composition (a) product purity; (b) impurity content.

**Fig. 8:**
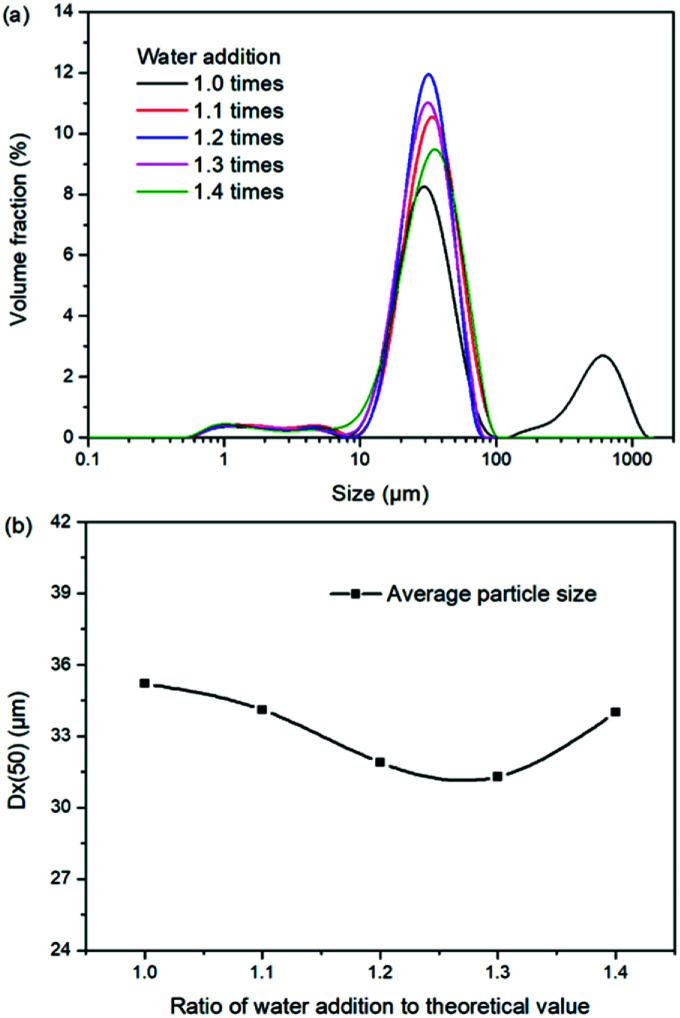
Effects of water addition on (a) crystal size distribution; (b) crystal averaged size.

Based on the results in Fig. 6 and 7, the main conclusion is that the water addition is an important factor to balance the product quality and resource recovery. As shown in Fig. 6, it is easy to understand that the liquid phase concentrations decrease with the increasing water addition due to the dilute effect. The more water addition, the more salts dissolved. Hence, the potassium recovery has an apparent decreasing trend. Meanwhile, Fig. 7 clearly shows that the product quality is improved by increasing water addition. The product has the characteristics of low purity and high impurity contents at the point of theoretical water addition. Especially the content of impurity Cl is significantly high. This is mainly attributed to the undissolved raw material, which also leads to the dual peaks of the crystal size distribution as shown in Fig. 8. In other words, because of the difference between the stable and metastable phase equilibrium, there is deviation in the calculation of the theoretical value so that the salt is not dissolved completely. When the water addition increases to 1.1 times of the theoretical value, the product quality has an apparent improvement. However, this effect becomes weaker and weaker when increasing the water addition further. Hence, it is not suggested to increase the water addition considering the decreased recovery and limited improvement of the product quality. In addition, as shown in Fig. 8, even if the crystal averaged size has the trend of decreasing first and increasing later, the general variation is insignificant. The minimum value 31.3 μm is closed to the maximum value 35.2 μm with the deviation ∼11%. Hence, water addition is an important control parameter for the indexes of product quality and resource recovery, whereas its effect on the crystal size is ignorable. In general, combing with the actual engineering operation, the optimized amount of water addition was recommended to be 1.1 times of the theoretical value to ensure the complete dissolution of the salts.

### Effects of agitation rate on the K_2_SO_4_ production

**Fig. 9:**
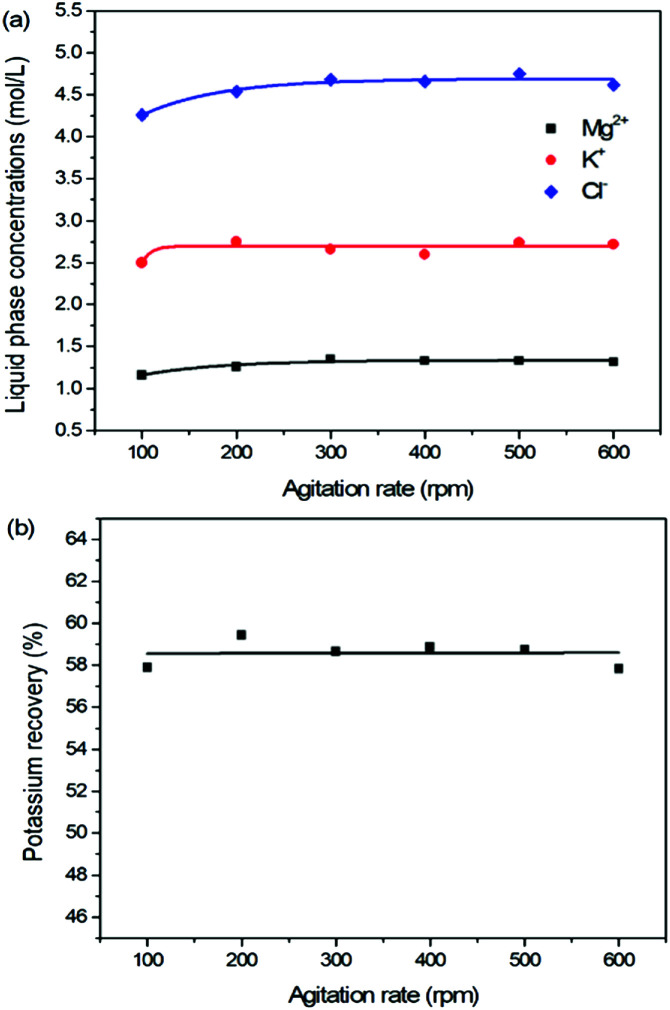
Effects of agitation rate on (a) liquid phase ion concentrations; (b) potassium recovery.

**Fig. 10:**
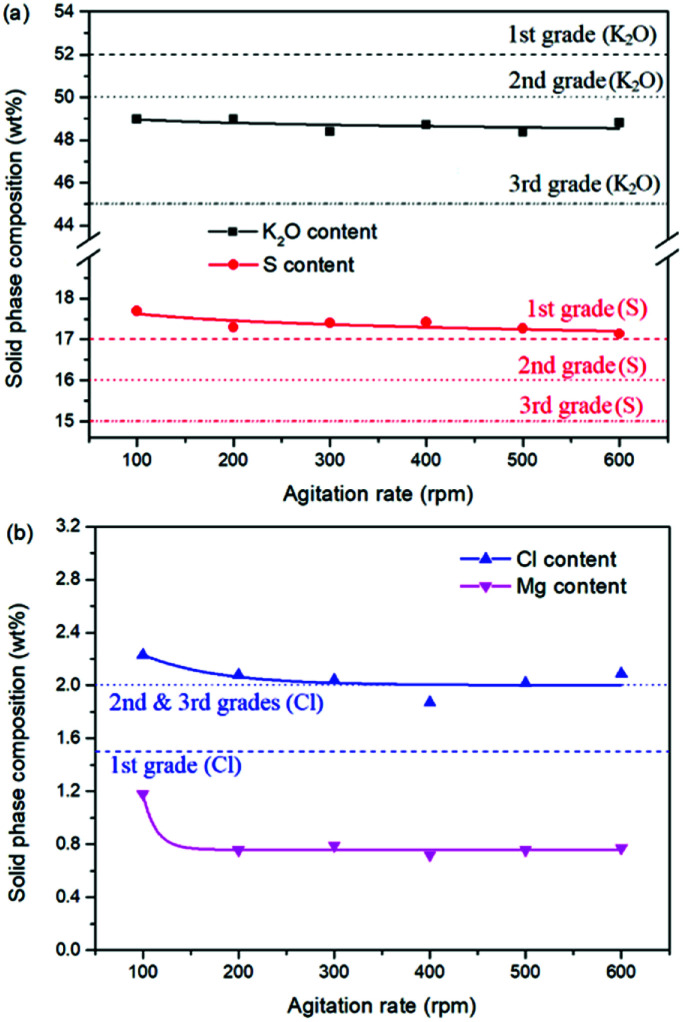
Effects of agitation rate on the product composition (a) product purity; (b) impurity content.

As one of the most important operating parameters in chemical engineering, the agitation rate was investigated by changing its value from 100 to 600 rpm. The other operating conditions were kept as leonite ore 50.0 g, KCl 32.0 g, deionized water 82.5 g, temperature 298.15 K. Corresponding results of the two-phase compositions are shown in Fig. 9 and 10. The results show that the two-phase components only have a slight variation at the beginning. When the agitation rate reaches 200 rpm, the composition results and potassium recovery are almost invariable with the product purity closed to the second grade of the national standard. This indicates that the process is mixing controlled at the low agitation rate of 100 rpm and the operating time of 30 min is not adequate to complete the reaction crystallization conversion. Improving the agitation rate is an effective way to eliminate the resistance of mixing diffusion. Hence, when the agitation rate increases, the reaction crystallization conversion equilibrium is almost independent on the mixing with the enough operating time of 30 min.

**Fig. 11:**
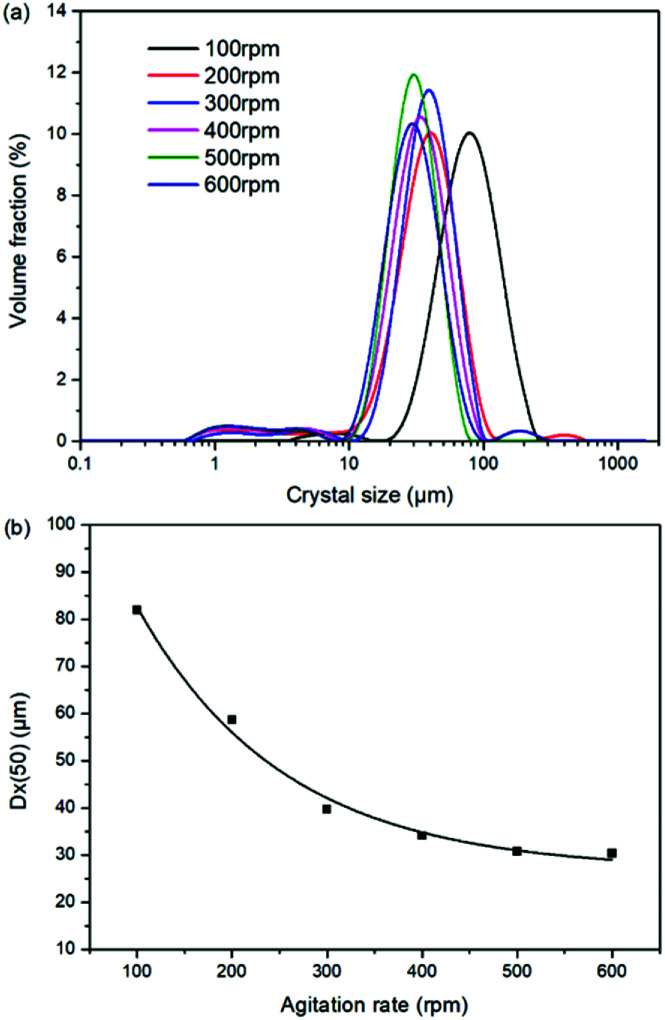
Effects of agitation rate on (a) crystal size distribution; (b) crystal averaged size.

As a kinetic parameter, the agitation rate has no significant effect on the K_2_SO_4_ product purity, however, its influence on the crystal size is non-negligible. As show in Fig. 11, the crystal size decreases apparently with the increasing agitation rate. The crystal averaged size reduced from 81.9 to 30.3 μm when the agitation rate increases from 100 to 600 rpm. This size variation is mainly ascribed to the effect of crystal breakage caused by the intensified stirring. Meanwhile, the decreasing trend is rapid at the beginning, while it becomes slower and slower gradually. Large crystal size is advantageous to the industrial filtration operation for reducing mother liquid entrainment. From this perspective, the agitation rate was suggested to be as small as possible on the preconditions of acceptable operating time to reach the equilibrium of the system. In the current operating case, the agitation rate of 200 rpm is suggested.

### Effects of operating temperature on the K_2_SO_4_ production

**Fig. 12:**
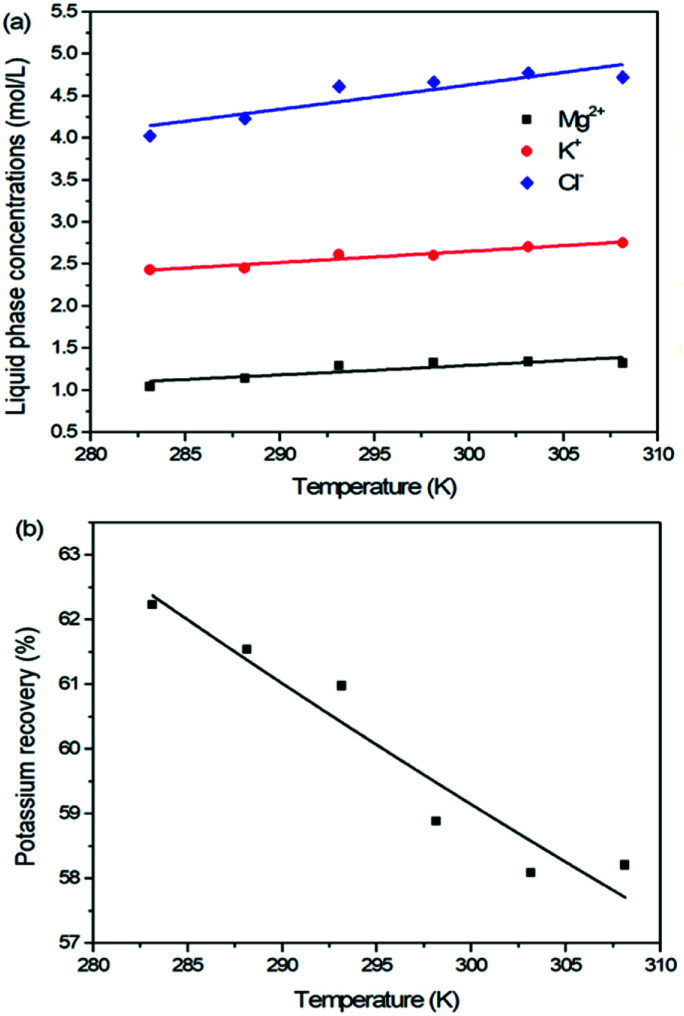
Effects of agitation rate on (a) liquid phase ion concentrations; (b) potassium recovery.

**Fig. 13:**
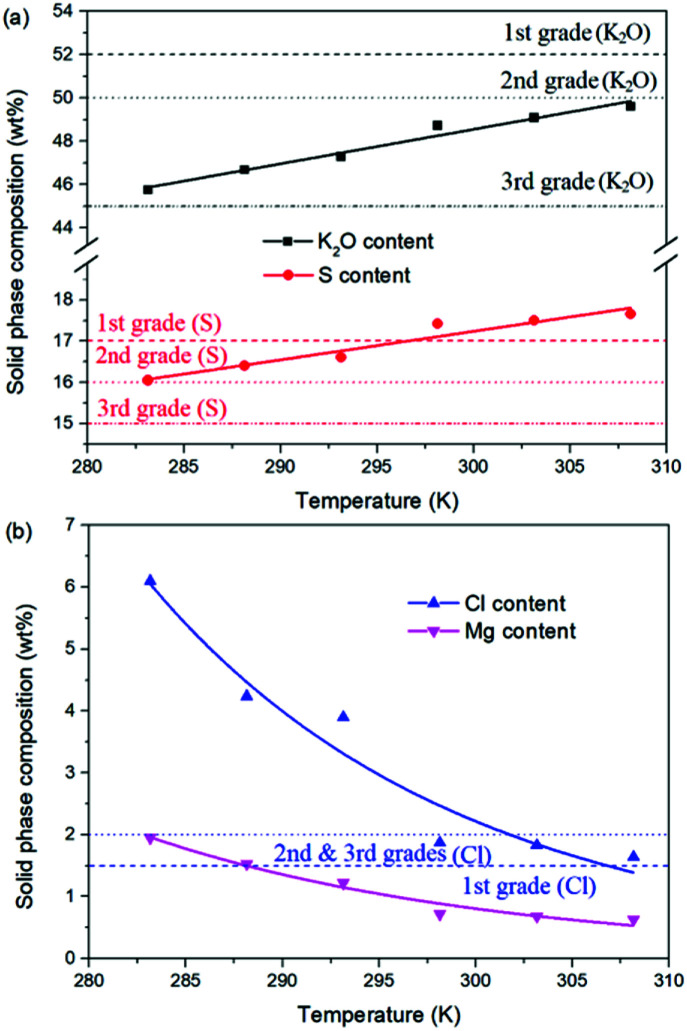
Effects of agitation rate on the product composition (a) product purity; (b) impurity content.

**Fig. 14:**
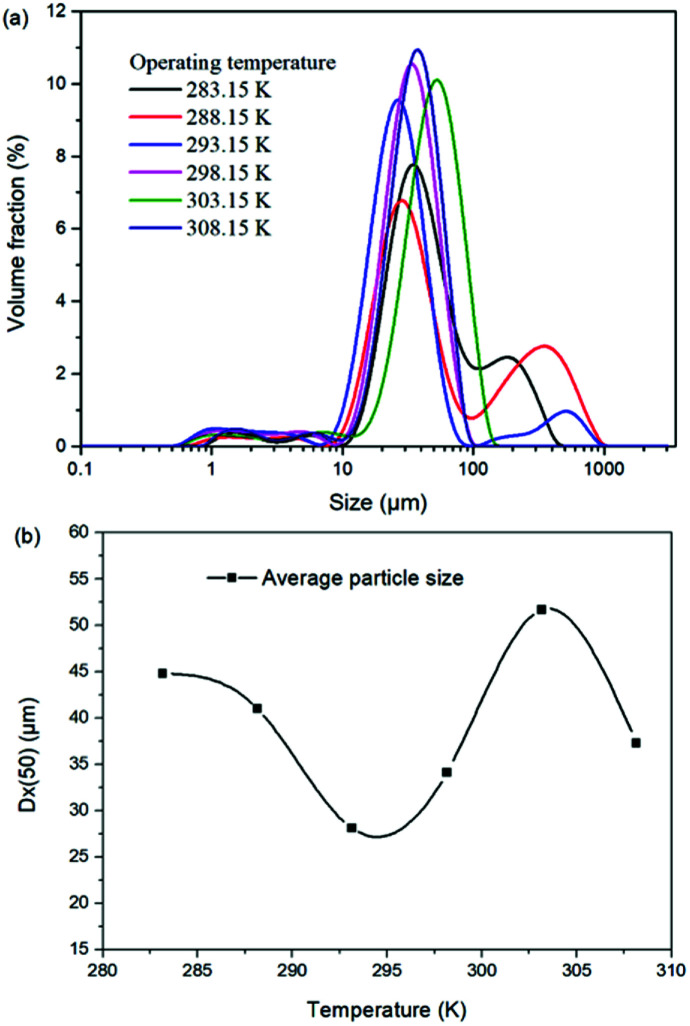
Effects of agitation rate on (a) crystal size distribution; (b) crystal averaged size.

For the reaction crystallization production of K_2_SO_4_, the operating temperature is an unavoidable topic for the process parameter investigation. Theoretically, expect the effects on the kinetics of dissolution, reaction and crystallization processes, the operating temperature may have significant influences on the product purity, yield and recovery due to the change of the system equilibrium. Hence, the K_2_SO_4_ production process was investigated at different temperatures of 283.15, 288.15, 293.15, 298.15, 303.15 and 308.15 K with the constant conditions of 400 rpm, 50 g leonite ore, 32 g KCl and 82.5 g deionized water. The corresponding variation trends of liquid phase concentrations, solid phase composition, potassium recovery and crystal size are shown in Fig. 12–14.

As shown in Fig. 12, the concentrations of Mg^2+^, K^+^ and Cl^−^ in the liquid phase increase gradually with the increasing operating temperature, namely, the liquid phase solubility and operating temperature have the positive correlation. Based on this fact, it can be considered as that the effect of operating temperature is similar to the one of water addition, because both of them influence the reaction crystallization process by changing the phase equilibrium regularity. Then, it is easy to understand that the potassium recovery is decreased because of the increased solubility at high temperature. Meanwhile, due to the variation of the solubility, the product quality is improved by increasing the temperature. It is clear that the contents of the main components K_2_O and S have a nearly linear increase with the operating temperature, while the impurities of Mg and Cl decrease apparently. Combing with the discussion in the section of water addition, the high contents of the impurities at low temperature is resulted from the undissolved KCl. This conclusion was further supported by the remarkable variation of the liquid phase Cl^−^ concentration and the dual peaks of the crystal size distributions as shown in Fig. 14. This is also the reason why the product impurities are reduced apparently when increasing the temperature from 283.15 to 293.15 K, whereas this improvement effect becomes weak when increasing the operating temperature further. From this perspective, the factors of operating temperature and water addition should be controlled synergistically to ensure the complete dissolution and conversion of KCl. Although the operating temperature and water addition have the same influence mechanism on the K_2_SO_4_ production, their effects on the crystal size distribution are various. The main difference is that the crystal averaged size decreases again when increasing the temperature from 303.15 to 308.15 K. This may be ascribed to that the high temperature accelerates the dissolution of KCl. Then, the supersaturation is increased so that the crystal size becomes small.

### Washing and refining of the crude K_2_SO_4_ product

The national standard GB/T 20406-2017 has the requirements of K_2_O > 52.0 wt%, S > 17.0 wt%, Cl < 2.0 wt% for the first grade product. Based on the previous parameter investigation, the crude K_2_SO_4_ product quality is closed to the second grade with its main limitation in the indexes of K_2_O and Cl contents. Usually, the mother liquor entrainment is the main factor, which influences the product purity directly. Hence, the washing and refining process of the crude K_2_SO_4_ product was focused in this section. With the operating conditions of 50.0 g leonite ore, 32.0 g KCl, 82.5 g deionized water, agitation rate 400 rpm and temperature 298.15 K, the dried crude K_2_SO_4_ product had the quantitative composition as shown in Table 2. The washing process was conducted for the wet crude K_2_SO_4_ before drying. The washing solution is saturated K_2_SO_4_ solution. The wet crude K_2_SO_4_ was mixed with the washing solution at the mass ratio of 2. Then, the refined K_2_SO_4_ product was acquired with the subsequent processes of solid–liquid separation and drying.

**Table 2:** Composition of the K_2_SO_4_ product

Items	Product composition (wt%)				Potassium recovery (%)
	K_2_O	S	Cl	Mg	
Dried crude product	48.73	17.42	1.87	0.72	58.88
Dried refined product	52.03	18.07	0.22	0.32	58.38
First rate (GB/T 20406-2017)	≥52	≥17.0	≤1.5	—	—

**Fig. 15:**
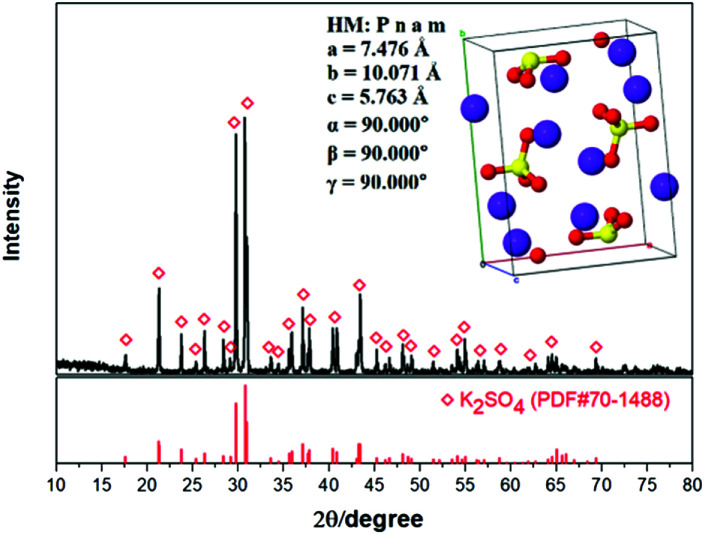
XRD pattern and crystal structure of washed K_2_SO_4_.

As shown in Table 2, the impurities were removed effectively by the washing process. This indicates that the existence of impurities Mg and Cl in the crude product is mainly attributed to the effect of mother liquor entrainment. Meanwhile, the product purity was improved from 48.73% to 52.03% correspondingly. Meanwhile, the XRD result shown in Fig. 15 indicates that the K_2_SO_4_ product has the orthorhombic structure with the cell parameters of *a* = 7.476 Å, *b* = 10.071 Å, *c* = 5.763 Å, *α* = *β* = *γ* = 90.000°. After washing process, the critical indexes of K_2_O, S and Cl meet the requirements of the first grade. Because of the use of saturated K_2_SO_4_ solution, the potassium recovery is almost constant with the acceptable value ∼58%. Related washing operation provides a reference for the actual industrial production.

## Conclusions

In this paper, the reaction crystallization production of K_2_SO_4_ was investigated for an optimal process operation to support the magnesium sulfate sub-type salt lake brine resource development in Western China. Based on the results of raw ore analysis and theoretical equilibrium calculation, the key process parameters were adjusted in specified ranges to clarify their effects on the indexes of product purity, potassium recovery and crystal size. The results show that the indexes of product quality and potassium recovery are critical for the parameter optimization of raw material ratio, water addition and operating temperature, whereas they are usually conflicting. The material mass ratio of leonite to KCl is suggested to be controlled for an equivalent reaction so that the raw materials can be converted completely with an optimized balance between the product quality and potassium recovery. For the parameters of water addition and operating temperature, they have the same effect mechanism on the K_2_SO_4_ production by changing the solubility equilibrium. The main operation principle is to ensure the complete dissolution of the raw materials. However, too much water addition and too high temperature should be avoided to intensify the dissolution, because the recovery will be decreased apparently with limited improvement of the product quality. By contrast, the effects of agitation rate are different. It is found that the product quality and recovery are almost independent on the agitation rate when the operating time is adequate, whereas the crystal size decreases significantly with the increasing agitation rate. Hence, small agitation rate is preferred for large size crystals provided the operating time is enough to ensure a non-mixing controlled production process. With the optimized operation conditions of raw material mass ratio 1.56 (leonite ore to KCl), 1.1 times of the minimum water addition, temperature 298.15 K, adequate agitation and a simple washing process, the K_2_SO_4_ product with the first grade quality (national standard GB/T 20406-2017) was acquired with the potassium recovery of 58.38%.

Related results provide an important operation guidance for the resource-type K_2_SO_4_ development in Qinghai. With reasonable equipment selection, it is expected to implement the technique for a true process in factory.

## Conflicts of interest

There are no conflicts to declare.

## Supplementary Material

## References

[cit1] Ciceri D., Manning D. A. C., Allanore A. (2015). Sci. Total Environ..

[cit2] Yan Z., Du S., Xie P., Shang L. (2018). Phosphate Compd. Fert..

[cit3] Grzmil B. U., Kic B. (2005). Chem. Pap..

[cit4] Sharma P. P., Yadav V., Rajput A., Kulshrestha V. (2018). ACS Omega.

[cit5] Han X. Z., Yan X., Wang X. Y., Ran J., Wu G. M., Zhang X. (2018). Sep. Purif. Technol..

[cit6] Sahare P. D., Moharil S. V. (1991). Radiat. Eff. Defects Solids.

[cit7] Xiong P. X., Peng M. Y. (2019). Opt. Mater. Express.

[cit8] Königsberger E., Eriksson G. (1999). J. Solution Chem..

[cit9] Abu-Eishah S. I., Bani-Kananeh A., Allawzi M. (2000). Chem. Eng. J..

[cit10] Trivedi J. S., Bhadja V., Makwana B. S., Jewrajka S. K., Chatterjee U. (2016). RSC Adv..

[cit11] Tomaszewska M. (2008). J. Membr. Sci..

[cit12] Tomaszewska M., Mientka A. (2009). Desalination.

[cit13] Mientka A., Grzmil B., Tomaszewska M. (2008). Chem. Pap..

[cit14] Li S. J., Song L. J. (2007). Inorg. Chem. Ind..

[cit15] Deng T. L., Yu X., Li D. C. (2009). J. Solution Chem..

[cit16] Mernagh T. P., Bastrakov E. N., Jaireth S., de Caritat P., English P. M., Clarke J. D. A. (2016). Aust. J. Earth Sci..

[cit17] Zheng M. P., Zhang Y. S., Liu X. F., Qi W., Kong F. J., Nie Z., Pu L. Z., Hou X. H., Wang H. L., Zhang Z., Kong W. G., Lin Y. J. (2016). Acta Geol. Sin..

[cit18] Chen Y. Z., Wang M. L., Yang Z. C., Liu C. L., Jiao P. C. (2001). Acta Geosci. Sin..

[cit19] Li H., Tang Z. F., Liu C. F., Lei G. Y. (2008). Acta Geosci. Sin..

[cit20] Li H., Liu C. F. (2008). Chemical Industry.

[cit21] Qin Z. (2016). Modern Salt and Chemical Industry.

[cit22] Liu C. F. (2009). Industrial Minerals & Processing.

[cit23] Li H., Chen Z., Lei G. Y. (2012). Adv. Mater. Res..

[cit24] Hu T. Q., Zhang Z. H., Wang J. K., Ma Y. F., Zhao D. M., Wang J., Zhang Y. M. (2017). Inorg. Chem. Ind..

[cit25] Zeng Z. M., Xia S. P., Hong X. L. (1994). J. Salt Lake Res..

[cit26] Song Y. H., Xia S. P. (1998). J. Salt Lake Res..

[cit27] Song Y. H., Xia S. P. (1995). J. Salt Lake Res..

[cit28] Song Y. H., Xia S. P. (1995). J. Salt Lake Res..

[cit29] Hu T. Q., Zhang Z. H., Dong O. Y., Zhao D. M., Ma Y. F., Fu Z. H., Wang J. K. (2016). J. Synth. Cryst..

[cit30] Hu T. Q., Zhang Z. H., Dong O. Y., Ma Y. F., Zhao D. M., Zhang Y. M., Wang J. K. (2017). J. Chem. Eng. Chin. Univ..

[cit31] Li S. J., Sun K. G., Zhao Y. L., Nie G. H., Song S. X. (2019). RSC Adv..

[cit32] Xiong P. X., Peng M. Y., Cao J. K., Li X. L. (2019). J. Am. Ceram. Soc..

[cit33] Xiong P. X., Peng M. Y. (2019). J. Mater. Chem. C.

